# Supplementary material for: Political factors and arrangements influencing primary health care financing and resource allocation: A scoping review protocol
Source: PLoS One. 2024 Aug 9;19(8):e0308754. doi: 10.1371/journal.pone.0308754 (PMC11315285; doi:10.1371/journal.pone.0308754)
Supplement: Attachment — Submitted filename: Response to reviewers.docx [file pone.0308754.s002.docx]

Dear academic editor and reviewers,

In return for PlosOne's observations raised during the review process, given the submission of the scoping review protocol entitled *"Political factors and arrangements influencing primary health care financing and resource allocation: a scoping review protocol"*, we are happy to present our responses to each point, as below:

1. Points raised by the academic editor

| **Point** | **Authors’ response** |
| --- | --- |
| 1. Please ensure that your manuscript meets PLOS ONE's style requirements, including those for file naming. The PLOS ONE style templates can be found at https://journals.plos.org/plosone/s/file  ?id=wjVg/PLOSOne  _formatting_sample_main_body.pdf  and https://journals.plos.org/plosone  /s/file?id=ba62/PLOSOne_formatting  _sample_title_authors_affiliations.pdf | The necessary adjustments were made, ensuring that the manuscript complies with Plos One guidelines for formatting titles, authors, and affiliations as well as the formatting of the main body. |
| 2. Thank you for stating the following financial disclosure:  “This work was supported by the FIOCRUZ Program for Strengthening Primary Health Care in Brazil – PMA 2019 (Fiocruz/VPPCb; grant number 25380.101539/2019- 05)”  Please state what role the funders took in the study. If the funders had no role, please state: "The funders had no role in study design, data collection and analysis, decision to publish, or preparation of the manuscript."  If this statement is not correct you must amend it as needed.  Please include this amended Role of Funder statement in your cover letter; we will change the online submission form on your behalf. | The funders had no role in the study protocol. Therefore, we inserted the suggested text in the Cover Letter: "The funders had no role in study design, data collection and analysis, decision to publish, or preparation of the manuscript." |
| 3. When completing the data availability statement of the submission form, you indicated that you will make your data available on acceptance. We strongly recommend all authors decide on a data sharing plan before acceptance, as the process can be lengthy and hold up publication timelines. Please note that, though access restrictions are acceptable now, your entire data will need to be made freely accessible if your manuscript is accepted for publication. This policy applies to all data except where public deposition would breach compliance with the protocol approved by your research ethics board. If you are unable to adhere to our open data policy, please kindly revise your statement to explain your reasoning and we will seek the editor's input on an exemption. Please be assured that, once you have provided your new statement, the assessment of your exemption will not hold up the peer review process. | As this is a research protocol, data and supporting information will be effectively generated, deposited and made available when we implement the proposed method (scoping review). In this sense, we will review the statement regarding data availability, which was previously stated when filling out the submission form. |
| 4. Please review your reference list to ensure that it is complete and correct. If you have cited papers that have been retracted, please include the rationale for doing so in the manuscript text, or remove these references and replace them with relevant current references. Any changes to the reference list should be mentioned in the rebuttal letter that accompanies your revised manuscript. If you need to cite a retracted article, indicate the article’s retracted status in the References list and also include a citation and full reference for the retraction notice. | We checked the 51 references cited throughout the manuscript and verified that they are compatible with the list of references presented at the end.  We didn’t identify citations of articles retracted in the manuscript |

2. Points raised by reviewer 1

| **Point** | **Authors' response** |
| --- | --- |
| “1. Although the research questions have been clearly stated, they could be further specified to better guide literature screening and data extraction. For example, it could be made clear which specific political factors and arrangements are the focus of this paper.” | We agree with the point regarding the research questions. Therefore, we have included a paragraph on page 6 that specifies and details political factors to guide study identification and data extraction.  *“The following political factors will be focused on: (1) at the governmental level, the composition of the government, its political and ideological orientations and priority health agendas; and (2) at the legislative level, the process related to economic and health regulation, involving the presence of lobbies, defence coalitions, legislative and party activities and representations of interests of different social groups.”*  We also changed Table 1 (p.7), to incorporate this detail into the key concept. |
| “2. The inclusion and exclusion criteria could be more detailed. It could be clarified which political factors (e.g. governmental system, fiscal policy, etc.) and resource allocation mechanisms (e.g.capitation, pay-for-performance, etc.).” | We agree that the inclusion and exclusion criteria need to be more detailed. Therefore, we made adjustments to two paragraphs on page 12, section 'Study Selection' (c), to explain more precisely the political factors defined as inclusion criteria and the financing and resource allocation mechanisms to be considered. Also, for this same reason, the content of Table 4 (p.13) was adjusted, both for the inclusion and exclusion criteria, to better identify the studies. |
| “3. While preliminary items for the data extraction table have been listed, they could be further refined. A breakdown of specific political factors and arrangements could be added, as well as specific indicators and outcomes related to primary health care financing and resource allocation.” | We made changes to Table 5 (pages 15 and 16), intending to refine the components guiding data extraction and analysis. We break down the last two items to highlight: (1) a list of thematic components referring to the political factors and arrangements considered, subdivided into “political and governmental systems” and “legislative environment”; and (2) a list of thematic components associated with indicators and outcomes regarding PHC financing and resource allocation |
| “4. In the methods section, the process of data extraction and analysis could be described in more detail, especially how to deal with the heterogeneity of multiple study designs and methods, and how to ensure consistency and accuracy of data extraction.” | We have adjusted the text of item d, “Data extraction,” specifying that data extraction will be organized by grouping the included references according to the sources of evidence to enable a more homogeneous extraction and analysis process of the reviewed material. Thus, we seek to reduce problems in extracting and analyzing information due to the heterogeneity of study designs and methods.  Regarding the consistency and accuracy of the data extraction process, we made significant adjustments to the text, highlighting the process's independent nature while defining the team of reviewers' constant review and improvement actions. Also, we incorporated a pilot test of filling out the data extraction form for necessary adjustments and corrections.  In Table 5, we repositioned items and defined a subsection of thematic data to guide the extraction process and, above all, the qualitative analysis in an inductive approach, considering concepts and context exposed in the PCC mnemonic.  These adjustments and deepening meet the recommendations presented by Pollock et al, 2023, already cited on protocol: Pollock, Danielle; Peters, Micah D.J.; Khalil, Hanan; McInerney, Patricia; Alexander, Lyndsay; Tricco, Andrea C.; Evans, Catrin; de Moraes, Érica Brandão; Godfrey, Christina M.; Pieper, Dawid; Saran, Ashrita; Stern, Cindy; Munn, Zachary. Recommendations for the extraction, analysis, and presentation of results in scoping reviews. JBI Evidence Synthesis 21(3):p 520-532, March 2023. \| DOI: 10.11124/JBIES-22-00123 |

3. Points raised by reviewer 2

| **Point** | **Authors' response** |
| --- | --- |
| “[…] some minor issues related to the **tentative schedule of data collection** and **some more clarification about the exact data analysis methods** and **how the results will be presented need to be mentioned** […]” | We have adjusted the review study schedule, planning to start selecting studies in November 2024 (p.11). We also include information that details the data analysis methods, reinforcing the role of the general descriptive analysis of the mapping and an inductive qualitative analysis, especially covering the themes expressed in the concepts and context derived from the research question (items ' d' and 'e' of section 2 'Methods and analyses').  We also inserted a few more elements to help us present the results, highlighting their association with the data extraction and analysis plan, refined in this manuscript review process. |

The marked changes are identified by a highlighted yellow colour in the revised version of the manuscript. The term 'table' was standardized (replacing the term 'box', present in the first version of the manuscript) for standardization and alignment with Plos One guidelines.

We hope to have fully complied with the recommendations and suggestions highlighted in the manuscript review. Thank you for your valuable contributions to our scoping review protocol.

Kind regards

Henrique S Dias

2024 July 12th
